# Epigenetic Silencing of Recombinant Adeno-associated Virus Genomes by NP220 and the HUSH Complex

**DOI:** 10.1128/jvi.02039-21

**Published:** 2022-02-23

**Authors:** Anshuman Das, Madhuvanthi Vijayan, Eric M. Walton, V. Grace Stafford, David N. Fiflis, Aravind Asokan

**Affiliations:** a Department of Surgery, Duke University School of Medicine, Durham, North Carolina, USA; b Department of Molecular Genetics & Microbiology, Duke University School of Medicine, Durham, North Carolina, USA; c Department of Biomedical Engineering, Duke University grid.471396.egrid.26009.3d , Durham, North Carolina, USA; University of California, Irvine

**Keywords:** AAV, DNA damage, gene silencing, gene therapy, virus-host interactions

## Abstract

The single-stranded DNA genome of adeno-associated viruses (AAV) undergoes second-strand synthesis and transcription in the host cell nucleus. While wild-type AAV genomes are naturally silenced upon integration into the host genome, recombinant AAV (rAAV) genomes typically provide robust expression of transgenes persisting as extrachromosomal DNA or episomes. Episomal DNA associating with host histones is subject to epigenetic modifications, although the mechanisms underlying such are not well understood. Here, we provide evidence that the double-stranded DNA binding protein NP220, in association with the human silencing hub (HUSH) complex, mediates transcriptional silencing of single-stranded as well as self-complementary rAAV genomes. In cells lacking NP220 or other components of the HUSH complex, AAV genome transcript levels are increased and correlate with a marked reduction in repressive H3K9 histone methylation marks. We also provide evidence that the AAV capsid (serotype) can profoundly influence NP220-mediated silencing of packaged genomes, indicating potential role(s) for capsid-genome or capsid-host factor interactions in regulating epigenetic silencing of rAAV genomes.

**IMPORTANCE** Recombinant AAV vectors can enable long-term gene expression in a wide variety of tissues. However, transgene silencing has been reported in some human gene therapy clinical trials. Here, we demonstrate the HUSH complex can suppress transcript formation from rAAV vector genomes by epigenetic modification of associated host histones. Further, the AAV capsid appears to play an important role in this pathway. We postulate that modulation of epigenetic pathways could help improve rAAV expression.

## INTRODUCTION

Prokaryotes and eukaryotes have evolved cellular defense mechanisms against “nonself” or foreign genetic material introduced by infectious agents. Unlike the widely studied CRISPR defense system in bacteria, mechanisms of silencing foreign DNA in eukaryotes are less well understood. Generally, eukaryotic cells rely on DNA sensors that recruit the host epigenetic machinery to label foreign DNA-bound histones with repressive methylation marks, resulting in inhibition of transcription (1). Although silencing foreign DNA presents an evolutionary advantage to the host over a pathogenic virus, this phenomenon could pose a significant challenge in the field of gene therapy.

Viral vectors such as adeno-associated virus (AAV) have emerged as popular gene delivery tools for treatment of many monogenic diseases. AAV is a small single-stranded DNA dependovirus in the *Parvoviridae* family, capable of delivering therapeutic transgenes into a wide range of tissues, resulting in long-lasting expression from episomes and with minimal risk of integration into the host genome (2). Despite recent success with FDA approval of AAV-based gene therapy for blindness and spinal muscular atrophy (SMA) (3), issues related to transient liver toxicity and gradual loss of transgene expression have been highlighted in some clinical trials for hemophilia and alpha-1 antitrypsin deficiency (4, 5). Although the loss of transgene expression has been demonstrated as a potential consequence of T-cell response against the AAV capsid or transgene product (6–9), the role of the vector genome and the flanking AAV inverted terminal repeats (ITRs) in eliciting a cellular silencing mechanism has not been studied in detail.

Recombinant AAV (rAAV) vectors contain a transgene between two inverted terminal repeats (ITRs) that serve as viral origin of replication and packaging signal (2). Further, it is known that most rAAV genomes undergo circularization and intermolecular recombination in the ITR region forming concatemers (10), which can persist for years as active episomes (11). Importantly, ITRs are key elements of the AAV genome presented to the foreign DNA-sensing machinery of the host cells. In this regard, the cellular DNA damage response (DDR) proteins play an important role in sensing rAAV genomes and modulating expression of the transgene. Specifically, ATM kinases and the MRN complex (Mre11/Rad50/Nbs1) interact with the ITRs and suppress concatemer formation and transgene expression (12–14). In contrast, some DNA repair proteins like DNA-PKcs and Ku70/80 directly bind to the ITRs by recognizing the ends as double-stranded breaks. This interaction promotes break-induced replication of rAAV genomes as well as concatamerization via the nonhomologous end-joining (NHEJ) repair pathway (15, 16). In addition to DDR, host proteins like the FKBP2 and RFX family of transcription factors were shown to bind the ITR D sequence, blocking second-strand synthesis and altering transgene expression (17). Another example is the KAP1 protein, which binds to rAAV genomes wrapped with histones and recruits the SETDB1 methyltransferase, resulting in deposition of repressive H3K9 marks (18). In addition, direct modification of the rAAV genomes, such as methylation of the CpG-rich regions in ITR and promoter of rAAV vectors, have been identified previously (19, 20), but direct mechanisms underlying these modifications remain unclear.

The human silencing hub (HUSH) complex was first identified as a multiprotein assembly of three proteins, MPP8, TASOR, and PPHLN1, involved in silencing of integrated HIV-1 and retroviral sequences called retroelements (21–23). Recent studies showed that integration-deficient murine leukemia virus (MLV) genomes undergo silencing by direct interaction with a double-stranded DNA binding protein called NP220, which, in turn, recruits the HUSH components and the methyltransferase SETDB1 to deposit repressive H3K9me3 histone marks (24). Interestingly, the same study also found that NP220 suppresses expression from unintegrated genomes of related retroviruses like HIV-1 and Mason-Pfizer monkey virus, but without any involvement of the HUSH complex (24). Since rAAV genomes do not undergo integration and mostly exist as extrachromosomal episomes, we evaluated whether the HUSH complex could play a similar role in regulation of rAAV gene expression. Specifically, in this study, we identified a novel role of NP220 and the HUSH complex in mediating transcriptional silencing of rAAV genomes by epigenetic modification of the associated histones. We also present evidence that the choice of AAV capsid (serotype) can profoundly influence these interactions and determine host-mediated silencing of packaged genomes.

## RESULTS

### Creation and validation of NP220 and HUSH complex knockout cell lines.

**FIG 1:**
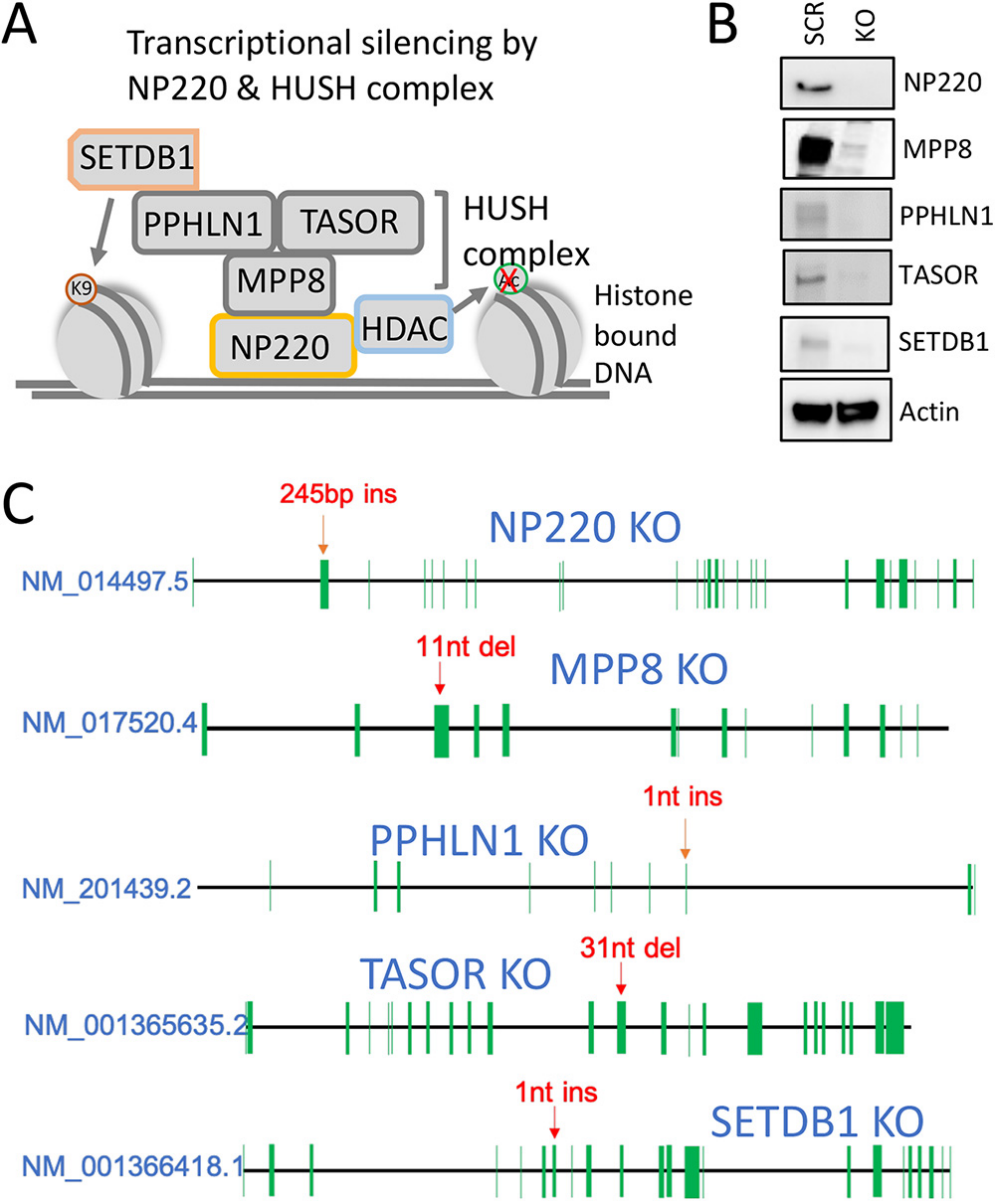
Generation and validation of NP220 and HUSH complex KO cells. (A) Schematic of HUSH complex recruited by NP220, which suppress transcription of both host and foreign genomes by binding to double-stranded DNA in regions of open chromatin. The HUSH complex recruits the methyl transferase SETDB1, which deposits repressive marks such as H3K9me3. The HUSH complex also recruits a histone deacetylase (HDAC), which removes activating acetyl marks. (B) Validation of HEK293 NP220 and HUSH KO cell lines by Western blotting. Left lane, scramble; right lane, knockout. Actin served as the loading control. (C) Schematic showing location of insertions (ins) and deletions (del) in the HEK293 NP220 and HUSH KO cell lines. Each green line represents an exon, and the position of each exon is not drawn to scale.

Double-stranded DNA of the host genome or foreign viral genomes is detected and bound by DNA sensor NP220, which, in turn, recruits the HUSH complex, comprised of MPP8, PPHLN1, and TASOR (Fig. 1A). The NP220-HUSH complex further recruits histone-modifying enzymes such as histone deacetylase (HDAC) and histone methyltransferase (SETDB1), which lead to removal of acetyl marks and addition of repressive methyl marks, respectively (Fig. 1A). This ultimately results in suppression of transcription (Fig. 1A). To understand the role of NP220 and HUSH complex members during AAV transduction, we generated knockouts (KOs) of NP220 and individual HUSH complex components (Fig. 1A) on HEK293 cell background using the CRISPR/Cas9 gene-editing technique. A single guide RNA (sgRNA) was used to target the Cas9 nuclease to a specific exon of target genes (Table 1). The polyclonal population of KO cells was selected in puromycin, from which single-cell clones were isolated for each KO and validated by Western blotting (Fig. 1B). Further, Sanger sequencing of the region upstream of the sgRNA target sequence in each clonal KO line showed indels of various lengths in the target exon (Fig. 1C), resulting in complete abrogation of the target protein expression (Fig. 1B).

**TABLE 1:** sgRNA sequences used in this study

NCBI gene ID	Gene name	sgRNA sequence (5′–3′)	Target exon
27332	NP220	CTTGAAGTACGTATTTATGA	2
54737	MPP8	GAGAGAGCAGGGCAGGACAT	3
51535	PPHLN1	AGGTGTTAGACAAACCCAGT	8
23272	TASOR	GGAAAACGAAATAACTCAAG	12
9869	SETDB1	AAGGAAAGAGTCTACTGTCG	7

### NP220 and HUSH complex components inhibit AAV transgene expression.

**FIG 2:**
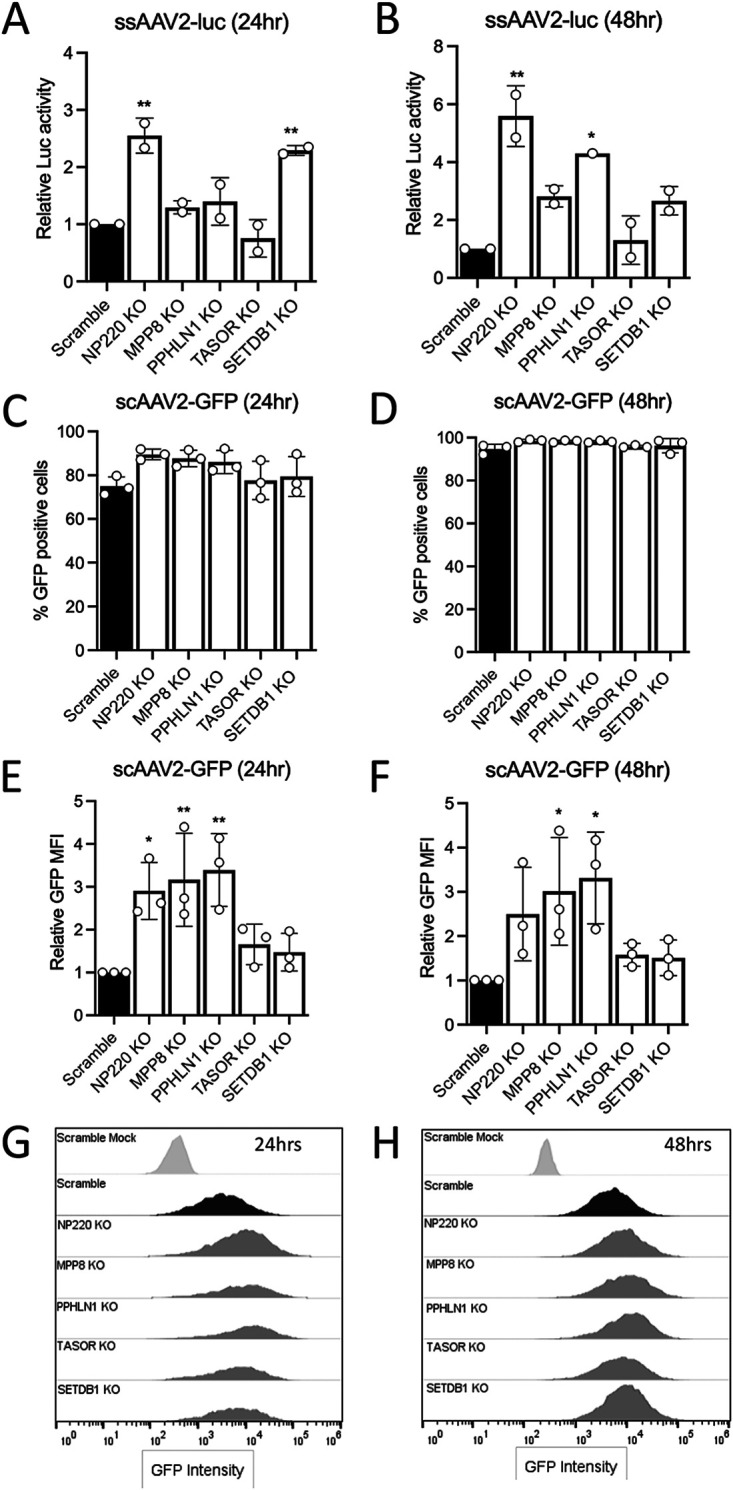
NP220 and the HUSH complex inhibit rAAV transgene expression. (A and B) Relative luciferase activity in HEK293 NP220 and HUSH KO cell lines transduced with AAV2 single-stranded (ss) luciferase virus at 5,000 vg/cell. Luciferase assay was performed at 24 and 48 h postransduction, and raw luciferase values were normalized to the scramble. (C and D) Percentage of GFP-positive HEK293 NP220 and HUSH KO cell lines transduced with AAV2 self-complementary (sc) GFP virus at 5,000 vg/cell. Flow sorting was performed at 24 and 48 h postransduction. (E and F) Relative GFP mean fluorescence intensity (MFI) of HEK293 NP220 and HUSH KO cell lines from flow cytometry experiment shown in panels C and D. Raw MFI values were normalized to the scramble. (G and H) Histograms of GFP mean fluorescence intensity (MFI) of HEK293 Scr and various KO cell lines shown in panels E and F. Scr mock represents untransduced cells. Data shown in panels A and B are from 2 independent experiments with 3 technical replicates each. Data shown in panels C to F are from 3 independent experiments with 2 technical replicates each. Error bars represent mean ± SD. Statistical significance between groups was calculated by one-way ANOVA with Dunnett’s multiple-comparison test. *, *P < *0.05; **, *P < *0.01.

To assess the effect of abrogating components of the HUSH complex on AAV transduction, we transduced the NP220 and HUSH complex KO cells with recombinant AAV2 packaging single-stranded luciferase transgene (ssAAV2-luc) and measured luciferase activity at 24 and 48 h. At both time points, a significant increase in luc activity was observed in NP220 KO cells compared to the scramble control (Scr) (Fig. 2A and B). Among the HUSH complex members, luc activity was significantly higher in SETDB1 KO at 24 h, whereas PPHLN1 KO was significantly higher at 48 h than Scr (Fig. 2A and B). We did not observe any difference in transduction of TASOR KO (Fig. 2A and B), likely due to growth defect resulting from the knockout (data not shown).

To evaluate the effect of NP220 and HUSH complex KO on transduction of AAV2 capsid packaging self-complementary green fluorescent protein (GFP) transgene, we tested the scAAV2-GFP transduction by flow cytometry. The percentage of GFP-positive cells did not change between Scr and various KO lines, except at 24 h, showing significantly higher transduction of NP220 KO cells (Fig. 2C and D). Similarly, the GFP mean fluorescence intensity (MFI) was significantly higher in NP220, MPP8, and PPHLN1 KO cell lines than in Scr cells (Fig. 2E to H), supporting a potential role for NP220 and the HUSH complex in inhibiting AAV transgene expression.

### NP220 inhibits AAV transgene expression at the transcriptional level.

**FIG 3:**
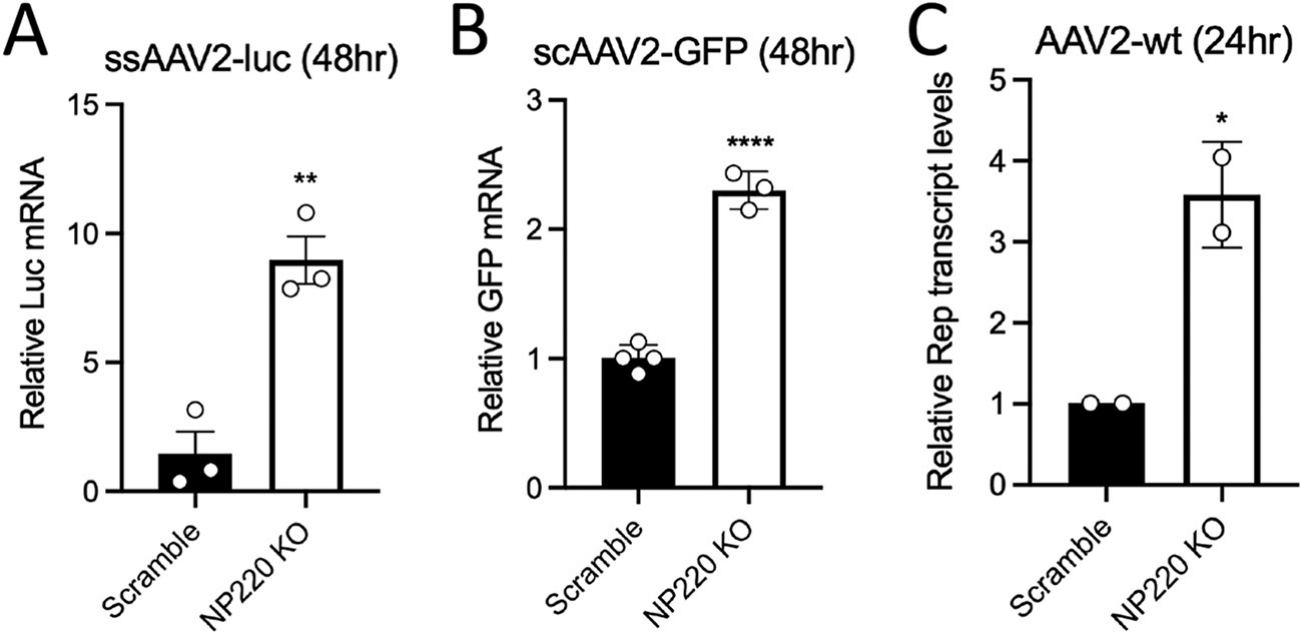
NP220 regulates rAAV and wtAAV genome transcription. (A) Relative Luc mRNA quantification by RT-qPCR of total RNA isolated from HEK293 Scr and NP220 KO cells transduced with ssAAV2-luc (5,000 vg/cell) at 48 h postransduction. (B) Relative GFP mRNA quantification by RT-qPCR of total RNA isolated from HEK293 Scr and NP220 KO cells transduced with ssAAV2-GFP (5,000 vg/cell) at 48 h postransduction. (C) Relative Rep transcripts quantified by RT-qPCR of total RNA isolated from HEK293 Scr and NP220 KO cells transduced with AAV2 wild type (10,000 vg/cell) at 24 h postransduction. The cells were transfected with 1 μg of pXX680 (adenovirus helper plasmid) 24 h prior to AAV2 wild type transduction. Data shown are from a representative experiment with two to three technical replicates each. Error bars represent mean ± SD. Statistical significance between groups was calculated by two-tailed unpaired *t* test. *, *P < *0.05;**, *P < *0.01.

Since NP220 plays a key role in silencing unintegrated viral genomes and NP220 KO consistently showed higher AAV transgene expression irrespective of the transgene and ITR configuration, we focused on the role of NP220 in silencing AAV transgene transcription. To this end, we used reverse transcriptase quantitative PCR (RT-qPCR) to measure the level of luciferase mRNA accumulated in Scr and NP220 KO cells 48 h after transduction with ssAAV2-luc and observed a significant increase (∼6- to 8-fold) in luc mRNA in NP220 KO cells (Fig. 3A). We also observed a modest increase in GFP mRNA (∼2.5-fold) in NP220 KO cells transduced with scAAV2-GFP (Fig. 3B), corroborating differences in transcription kinetics of single-stranded versus self-complementary vector genomes (25). Nevertheless, these transcription data correlate well with the luciferase activity and GFP fluorescence data shown in Fig. 2. Finally, we evaluated transcription of wild-type AAV2 genomes in Scr and NP220 KO cells by quantifying Rep transcript levels using RT-qPCR. At 24 h postransduction, we observed ∼3- to 4-fold increase in Rep transcripts produced from the viral p5 and p19 promoters in NP220 KO cells (Fig. 3C), suggesting both wild-type and recombinant AAV genomes are subject to NP220-mediated suppression of transcription.

### NP220 restricts expression of transgene delivered by diverse AAV serotypes.

**FIG 4:**
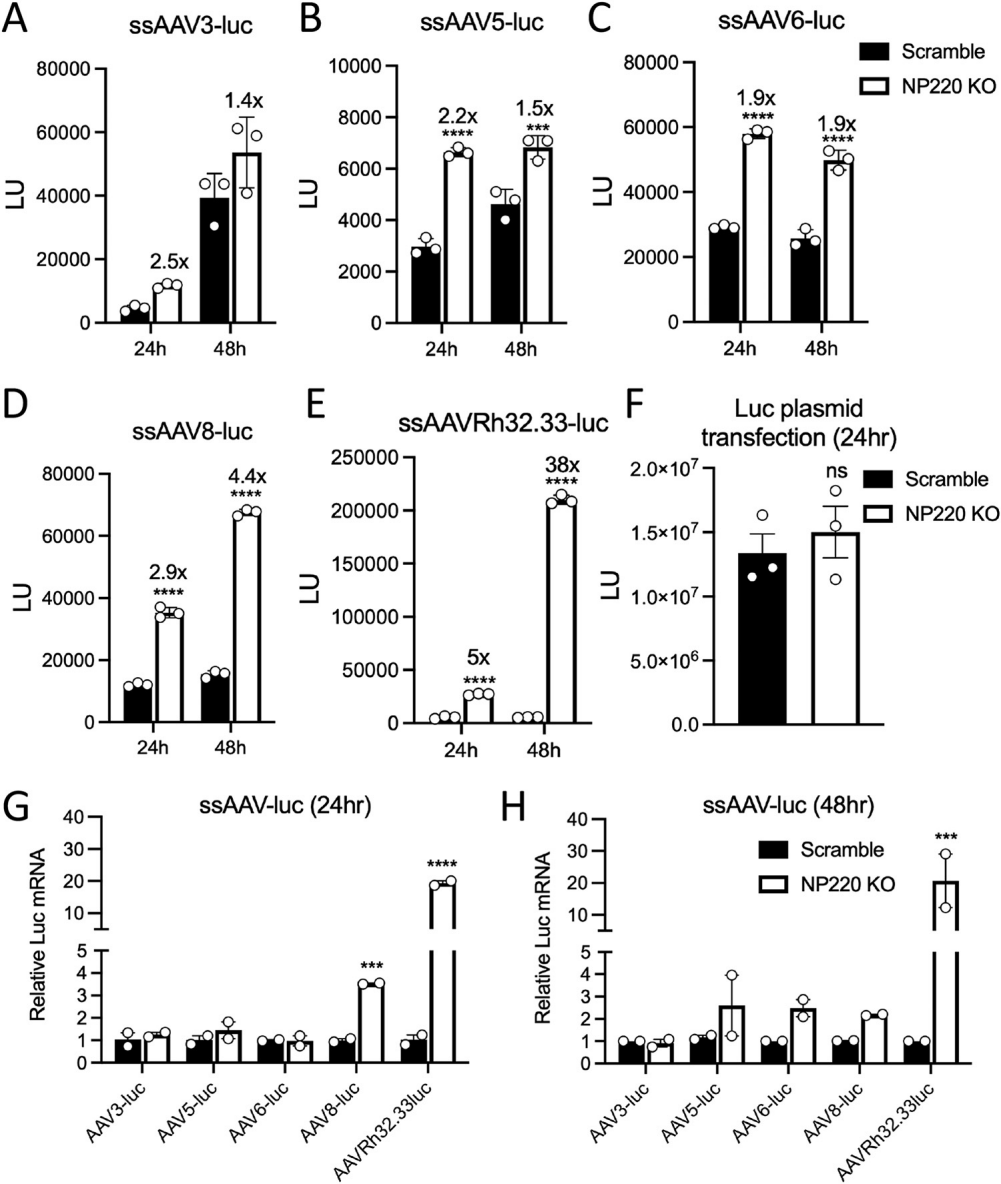
NP220 restricts expression of transgene delivered by diverse AAV serotypes. (A to E) Luciferase activity in HEK293 Scr and NP220 KO cells transduced with indicated AAV capsids packaging a single-stranded (ss) luciferase genome at 10,000 vg/cell. Luciferase assay was performed at 24 and 48 h postransduction. Raw luciferase light units are plotted on the *y* axis. Data shown are three technical replicates from a representative experiment performed twice independently. (F) Luciferase activity in HEK293 Scr and NP220 KO cells transfected with a plasmid expressing luciferase under chicken beta-actin (CBA) promoter flanked by AAV ITRs. Luciferase assay was performed at 24 h postransduction. Data shown are three technical replicates from a representative experiment. (G to H) Relative luciferase mRNA quantification by RT-qPCR of total RNA isolated from HEK293 Scr and NP220 KO cells transduced with indicated AAV capsids packaging a single-stranded (ss) luciferase genome transduced at 10,000 vg/cell. Data shown are two technical replicates from a representative experiment performed twice independently. Error bars represent mean ± SD. Statistical significance between groups was calculated by two-way ANOVA with Sidek’s multiple-comparison test. *, *P < *0.05; **, *P < *0.01.

We expanded our analysis to other naturally occurring AAV serotypes. To this end, Scr and NP220 KO cells were transduced with various AAV serotypes packaging a single-stranded luciferase transgene, and luciferase activity was measured at 24 and 48 h postransduction. When transduced with 10,000 vector genomes (vg)/cell of AAV 3-, 5-, 6-, or 8-luc, we observed a ∼2- to 4-fold increase in luciferase activity in NP220 KO cells compared to the Scr (Fig. 4A to D), while AAV Rh32.33-luc transduction was enhanced by up to 38-fold after 48 h (Fig. 4E). These data are consistent with luciferase mRNA levels quantified by real-time PCR, which showed the largest increase in luciferase genome transcription in the case of the AAVrh32.33 capsid (Fig. 4G and H). Finally, to test if the capsid played an essential role in NP220-mediated suppression, we transfected a plasmid encoding luciferase in Scr and NP220 KO cells and observed no difference in luciferase expression (Fig. 4F). Taken together, these data suggest that host factor interactions with AAV capsids or capsid-genome interactions that might determine postentry steps could influence epigenetic repression imposed by NP220 and the HUSH complex.

### Epigenetic modification of the AAV genome is regulated by NP220.

**FIG 5:**
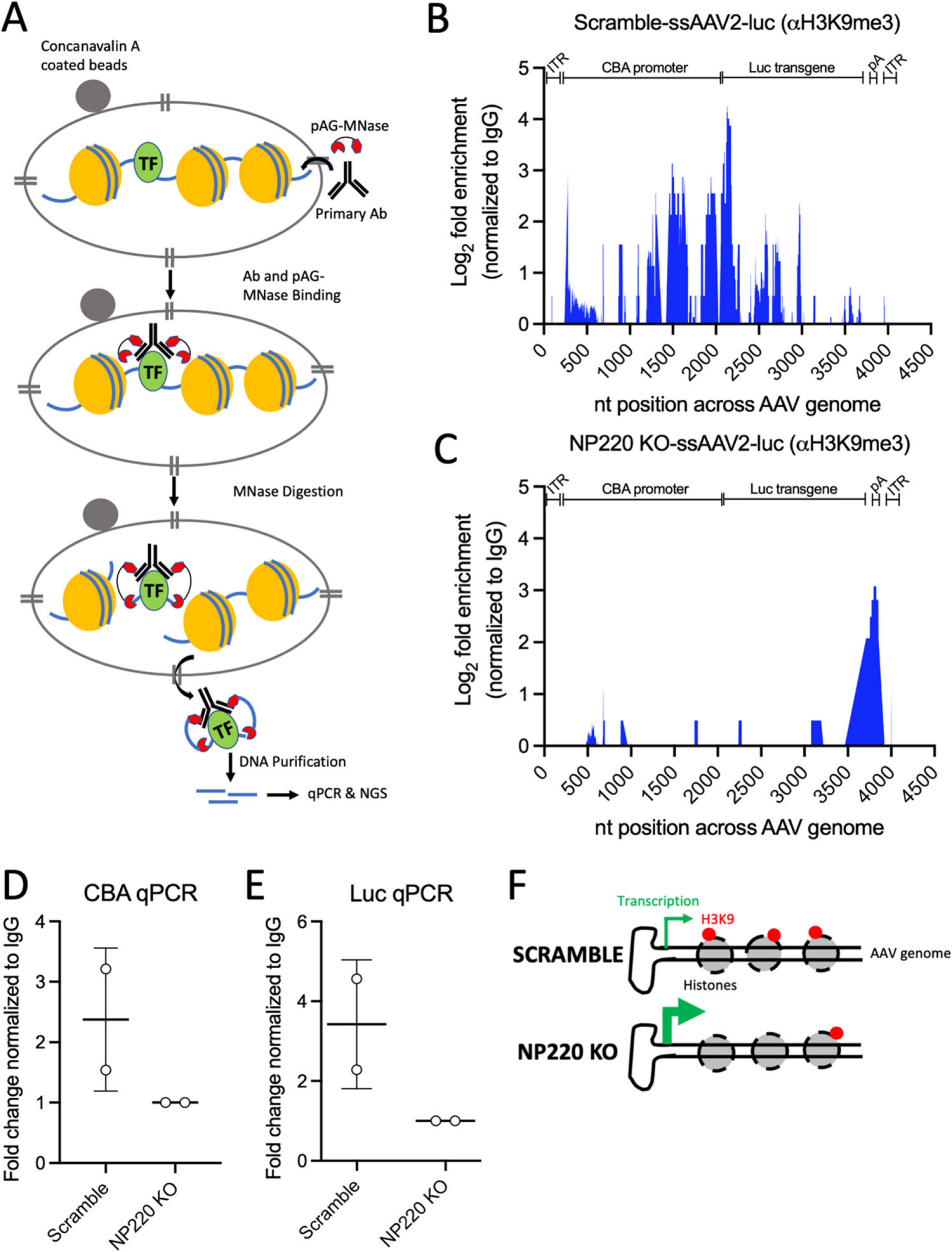
Epigenetic modification of AAV genomes is regulated by NP220. (A) Schematic of Cut&Run assay, a highly sensitive version of ChIP assay, used here to determine presence of methylation marks on histones bound to the AAV genome. (B and C) Mapping of reads to the AAV genome obtained from next-generation sequencing of Cut&Run assay H3K9me3 immunoprecipitates from HEK293 Scr (B) and NP220 KO cells (C) transduced with ssAAV2-luc at 1,000 vg/cell for 72 h. Percentage of reads mapped to the AAV genome in H3K9me3 antibody pulldown samples were subtracted from matched isotype IgG antibody pulldown samples and expressed as log_2_ fold enrichment normalized to IgG. (D and E) qPCR quantification of AAV DNA sequences mapping to the peaks observed in panel B. Specific primers targeting regions in the CBA promoter (D) and luciferase transgene (E) were used in the qPCR. Fold change in the threshold cycle (*C_T_*) values is normalized to the IgG control. Data shown are from two independent experiments. Error bars represent mean ± SD. (F) Schematic depicting the negative regulatory role of NP220 on AAV gene expression. Enhanced transcription of AAV genome is observed in cells lacking NP220 (KO), whereas cells expressing NP220 (Scr) suppress AAV gene expression by depositing repressive H3K9me3 marks across the AAV genome, especially in the promoter and transgene.

It is well established that NP220 and the HUSH complex are recruited to DNA-bound histones with H3K9 methyl marks, where they further recruit SETDB1 to maintain the H3K9-repressive marks (24). Since we showed here that NP220 and the HUSH complex regulate AAV vector genome transcription, we wanted to further evaluate if epigenetic modifications are responsible for increased AAV genome transcript levels in NP220 KO cells. To this end, we performed a modified chromatin immunoprecipitation (IP) called a Cut&Run assay, which involves IP with an antibody specific for the H3K9me3 mark, followed by micrococcal nuclease digestion of DNA bound by the target antibody (Fig. 5A). The DNA fragments released are purified and subjected to real-time PCR and next-generation sequencing (NGS) (Fig. 5A). The Cut&Run assay was performed on scramble and NP220 KO cells transduced with a multiplicity of infection (MOI) of 1,000 for ssAAV2-luc for 72 h. To evaluate the abundance and distribution of the H3K9me3 marks across the AAV genome, we subjected the H3K9me3 and control IgG-IP DNA from the Cut&Run assay shown above to next-generation Illumina sequencing. The reads were aligned to the AAV genome, and the number of reads identified at a given position in scramble and NP220 KO samples was subtracted from the respective IgG control samples (Fig. 5B and C). We observed ∼2- to 4-fold enrichment in H3K9me3-marked DNA in the scramble cells compared to NP220 KO, particularly around the GC-rich regions in the promoter and the transgene (Fig. 5B and C). A distinct peak in the poly(A) region downstream of the luciferase transgene was observed in NP220 KO (Fig. 5C), although its relevance is not clear at this time. To further confirm that the peaks observed in Fig. 5B are not a result of sequencing bias, we used the H3K9me3 and control IgG-IP DNA to perform qPCR using primers specific to sequences around the peak and observed ∼2- to 4-fold enrichment of AAV sequences in Scr compared to NP220 KO (Fig. 5D and E). Overall, these data point to an important role of NP220 in epigenetic silencing of AAV genomes. Based on these data, we propose a scenario wherein NP220 and the HUSH complex are actively involved in suppressing transcription of AAV genomes’ H3K9 methylation of associated histones. In the absence of components of the NP220-HUSH repressor complex, transcription of AAV genomes is enhanced due to lack of H3K9 methylation, resulting in higher transgene expression (Fig. 5F).

## DISCUSSION

Eukaryotic cells employ epigenetic mechanisms to modulate endogenous gene expression and, in the case of invading pathogens, act as a defense mechanism by suppressing foreign genes. In the case of retroviruses that integrate into the host genome or recombinant AAV genomes that exist as episomes, both are subjected to epigenetic silencing (18, 22). NP220 was recently shown to interact with the long terminal repeats (LTRs) of unintegrated genomes of murine leukemia virus (MLV) and recruit the HUSH complex to impart repressive H3K9me3 marks, thereby effectively shutting down viral transcription (24). Recombinant AAV genomes that typically exist as unintegrated episomes bound to histones have been shown to undergo DNA hypermethylation resulting in reduced gene expression (19, 20). Here, we show that NP220 and the HUSH complex regulate AAV gene expression by depositing repressive marks on the bound histones.

Of the different components of the HUSH complex that were evaluated, the knockout of NP220 had the greatest impact on AAV transgene (luciferase) expression. Interestingly, luciferase expression in NP220 KO gradually increased from 24 to 48 h compared to Scr cells, suggesting more genomes were transcribed and translated in the absence of NP220. This points to a key role of NP220 in regulating epigenetic silencing of AAV vector genomes, but it also raises the possibility that NP220 might recruit additional histone-modifying factors other than the HUSH complex, as previously observed for HIV-1 and Mason-Pfizer monkey virus (24). Interestingly, NP220 is highly conserved across vertebrate species, but the proteins that act in concert with NP220 to suppress viral gene expression can differ depending on the virus and the host species (26).

Recombinant AAV genome configuration, whether single-stranded or self-complementary, did not seem to affect NP220-mediated suppression of transgene expression. However, AAV capsid serotype packaging recombinant genomes profoundly impacted transgene expression in NP220 KO cells. While transduction of NP220 KO cells with most natural AAV isolates we tested showed ∼2- to 3-fold enhancement in luciferase transgene expression, the AAVRh32.33 capsid displayed ∼20- to 40-fold increase in luciferase expression. This observation is particularly intriguing given the robust immunogenicity of AAVRh32.33 observed in several animal models (27, 28). Currently, the exact mechanism by which AAVRh32.33 delivered genomes appears (hyper)sensitive to NP220-mediated suppression of transgene expression is unclear. Whether this effect arises from capsid-genome interactions specific to AAVRh32.33 or the ability of the capsid to recruit DNA-sensing host factors remains to be determined. Nevertheless, the role of the capsid is evident since transfected DNA carrying the luciferase transgene flanked by AAV ITRs (bypassing entry) was not subject to silencing. These observations are further bolstered by recent evidence presented by our group and others affirming the role of the AAV capsid in second-strand synthesis of single-stranded genomes and subsequent vector genome transcription (29–32).

The mechanism of NP220- and HUSH complex-mediated regulation of retro- and lentiviral gene expression is well understood, primarily occurring through deposition of repressive H3K9me3 marks on the bound histones (24, 33). Using the sensitive Cut&Run assay combined with next-generation sequencing, we found that AAV genomes delivered into scramble cells had highly enriched H3K9me3 marks compared to NP220 KO, and most of these marks were localized to GC-rich sequences in the ITR, promoter, and parts of the transgene. Since NP220 is known to bind to the consensus sequence 5′-CCCCCG/C-3′ (34), it might possibly explain why GC-rich sequences were present within ∼50 to 100 bp of the peaks observed in our analysis. These GC stretches might allow binding of NP220 and recruitment of the HUSH complex (24, 34). It will be interesting to evaluate if modifying these GC-rich sequences allows AAV genomes to escape NP220-mediated suppression and potentially lead to higher transgene expression; however, this approach is also likely to affect packaging and other functions associated with the ITRs and warrants extensive investigation. Indeed, certain retroviruses like Rous Sarcoma virus (RSV), which has low cytidine content in the LTR sequences, do not respond to NP220-mediated silencing (24), suggesting NP220 preferentially binds to cytidine-rich viral sequences. Another approach can involve inhibition of host-silencing mechanisms by small-molecule histone deacetylase (HDAC) inhibitors, which have been shown to enhance AAV transgene expression in animal tumor models (35, 36). The impact of HUSH complex-mediated silencing *in vivo* remains to be determined and can plausibly be modeled in KO mouse models where the corresponding molecular components are lacking or potentially through pharmacological modulation in preclinical gene therapy applications. In addition, whether NP220 and the HUSH complex or other independent mechanisms operate in different cell types *in vivo* and consequently impact gene therapy applications utilizing different AAV serotype vectors are critical topics for future investigation.

In summary, this study presents an initial body of evidence supporting the role of NP220 and HUSH complex in negatively regulating AAV transgene expression by epigenetic silencing. Future studies evaluating transcriptional factors and host-silencing mechanisms in different animal models could offer additional valuable insight into AAV biology as well as provide a roadmap for devising strategies to address transgene silencing in related gene therapy applications.

## MATERIALS AND METHODS

### Cell lines.

Human embryonic kidney (HEK293) cells were obtained from the University of North Carolina Vector Core. Cells were maintained in Dulbecco’s modified Eagle medium (DMEM) supplemented with 10% fetal bovine serum (FBS), 100 U/mL penicillin, and 100 μg/mL streptomycin and were grown in 5% CO_2_ at 37°C. For generation of NP220 and HUSH KO lines, HEK293 cells were seeded on 6-well plates and incubated with media containing the recombinant lentivirus (encoding either the scrambled guide control [Scr] or the CRISPR guide targeting NP220 and individual HUSH factors). Cells were spinfected with the recombinant lentivirus for 30 min at 400 × *g* at room temperature. One day after spinfection, the medium was replaced with fresh media, and 2 days after transduction, cells were maintained in selection medium containing puromycin for 7 days. Following selection, clonal lines were generated by isolating single clones of cells and amplifying them for knockout validation by Western blot and sanger sequencing analyses. Guides were ordered as single-stranded oligonucleotide sequences from IDT. The single guide RNA (sgRNA) sequences (5′ to 3′) that were cloned into LentiCRISPRV2 are shown in Table 1.

### Recombinant and wild-type AAV vector production.

Recombinant AAV vectors packaging a single-stranded luciferase transgene driven by a chicken β-actin (CBA) promoter or a self-complementary GFP transgene driven by a chicken β-actin-minute virus of mice (MVM) intron hybrid (CBH) promoter (scAAV-GFP) were generated using the standard triple-plasmid transfection system in HEK293 cells as described previously (37). Depending on the serotype, cell lysates or supernatants or both were collected at 4 days and 7 days posttransfection and subjected to precipitation with 10% polyethylene glycol (PEG) overnight. The PEG pellet was resuspended in 1× phosphate-buffered saline (PBS) containing 2 mM MgCl_2_ and 0.001% F-68 (Gibco) and loaded on a 17 to 40% iodixanol gradient for ultracentrifugation at 30,000 × *g* for 16 to 20 h. Fractions of 500 μL were manually collected at the 32% to 40% interface, treated with DNase (0.1 mg/mL) for 1 h at 37°C, and subjected to qPCR. The fraction containing the peak titer was buffer-exchanged using Zeba Spin columns (Thermo Scientific) and eluted in 1× phosphate-buffered saline (PBS) containing 2 mM MgCl_2_ and 0.001% F-68. The eluted virus titer was again quantified by qPCR. Primers used for AAV titers bound to the AAV2 inverted terminal regions (ITRs) and the sequences are shown in Table 2.

**TABLE 2:** Primers used in this study

Primer name	Sequence (5′–3′)	*T_m_*
ITR-F	AACATGCTACGCAGAGAGGGAGTGG	73
ITR-R	CATGAGACAAGGAACCCCTAGTGATGGAG	72
GFP-F	AGTGCTTCAGCCGCTACCC	71
GFP-R	GTTGTACTCCAGCTTGTGCC	66
Luc-F	AAAAGCACTCTGATTGACAAATAC	61
Luc-R	CCTTCGCTTCAAAAAATGGAAC	62
P5/P19-F	GAGGACCAGGCCTCATAC	57
P5/P19-R	CTTTCCCGCATTGTCCAAGG	59
GAPDH-F	ACGGATTTGGTCGTATTGGG	65
GAPDH-R	TGATTTTGGAGGGATCTCGC	65
CutnRun-CBA-F	GCGTGCGTGTGTGTGTG	61
CutnRun-CBA-R	CTGCGGAGCGCACAAAG	60
CutnRun-Luc-F	GCTGGAGAGCAACTGCATAA	57
CutnRun-Luc-R	GTGATGTCCACCTCGATATGTG	57

AAV2 wild-type (WT) virus was produced similarly to rAAV2, except the ITR-transgene plasmid was replaced with wild-type plasmid containing full-length Rep and Cap genes flanked by ITRs. Virus harvesting and purification was done similarly to rAAV2. For transduction, Scr and NP220 KO cells were transduced with 10,000 vg/cell of AAV2 WT virus, and Rep transcripts were measured at 24 h postransduction by RT-qPCR using primers listed in Table 2.

### Luciferase transduction assays.

Cells were counted and seeded in 24-well plates (1 × 10^5^ cells per well) and allowed to adhere overnight. After 24 h, cells were transduced with various AAV capsids packaging a single-stranded genome expressing a luciferase transgene at the indicated MOI. Unless otherwise indicated, cells were harvested at 24 and 48 h after transduction in passive lysis buffer. The cell lysate was then combined with luciferin substrate from Promega. Luciferase signal was then quantified by a VictorX plate reader (PerkinElmer).

### RNA extraction and qPCR analysis.

Total RNA was isolated using the Quick RNA miniprep kit from Zymo Research, and extracted RNA was then reverse transcribed using the iScript cDNA synthesis kit (Bio-Rad) as per the manufacturer’s instructions. RT-qPCR was performed in triplicates using the Power SYBR green PCR master mix (Thermo Fisher) and the CFX96 Touch real-time PCR detection system (Bio-Rad). Primers targeting GFP, luciferase, and the internal control GAPDH are shown in Table 2.

### Western blotting and antibodies.

Cells were lysed in radioimmunoprecipitation assay (RIPA) lysis and extraction buffer containing protease inhibitor for 20 min. The lysate was clarified by centrifugation at 4°C for 10 min at 12,000 rpm. The samples were boiled at 95°C in SDS sample buffer containing β-mercaptoethanol and resolved by SDS-PAGE electrophoresis. The resolved proteins were transferred to a polyvinylidene difluoride (PVDF) membrane using the Trans-Blot Turbo semidry transfer system (Bio-Rad), and the membrane was blocked in 1× PBS containing 0.1% Tween 20 and 5% skim milk powder for 1 h at room temperature. The membranes were probed with specific primary and secondary antibodies. The primary antibodies used were mouse anti-actin (catalog no. ab3280) and PPHLN1 (catalog no. ab69569), which were purchased from Abcam. SETDB1 (catalog no. 11231-1-AP) and MPP8 (catalog no. 16796-1-AP) were purchased from Proteintech. ZNF638/NP220 (catalog no. A301-548A) was purchased from Bethyl Laboratories; TASOR (catalog no. HPA006735) was from Sigma-Aldrich. Goat anti-rabbit-horseradish peroxidase (HRP) (catalog no. 111-035-003) was obtained from Jackson ImmunoResearch. Goat anti-mouse-HRP (catalog no. 32430) was obtained from Thermo Fisher.

### Flow cytometry analysis.

About 1 × 10^5^ scrambled, NP220, and HUSH KO cells were transduced with AAV2-sc-GFP at 200, 1,000, or 5,000 vg/cell. At either 24 or 48 h postransduction, cells were dissociated in TripLE (Gibco), washed in PBS, resuspended in flow media (DMEM supplemented with 1% bovine serum albumin (BSA), 10 units/μL DNase, and 1% penicillin-streptomycin), and passed through a 100-μm cell strainer. Fluorescent intensity was measured by a Sony SH800 cell sorter following excitation with a 488-nm laser and analyzed using FlowJo. Briefly, cells were gated first on forward scatter height versus side scatter height, followed by gating for single cells identified by a linear correlation between forward scatter area versus forward scatter height.

### Cut&Run assay.

About 1 × 10^5^ scrambled and NP220 KO cells were mixed with ssAAV2-luc at 1,000 vg/cell, seeded in a 24-well plate, and incubated for 24 h at 37°C. The next day, virus inoculum was removed, and fresh medium was added and incubated for another 72 h at 37°C. This long incubation was intended to allow the majority of the AAV capsids to uncoat in the nucleus, thereby exposing the maximum number of AAV genomes to NP220 and the HUSH complex. Cells were trypsinized and counted, and each well was divided into 5 samples (IgG IP, NP220 IP, H3K4 IP, and H3K9 IP and Input) with 100,000 cells each. The rest of the Cut&Run assay was performed as per the manufacturer’s protocol. DNA from both IP and input chromatin samples was purified by spin columns (Invitrogen). Real-time PCR and NGS library preparation were done following the manufacturer’s protocol. The NGS library was sequenced on an Illumina HiSeq platform with a 2 × 150 configuration. The raw sequencing reads were analyzed by trimming the Illumina adapter sequences from the forward reads using the Cutadapt program (38) and retaining only reads with a length of ≥30 bp. A cutoff of length 30 was chosen based on manufacturer guidance for minimum fragment length generated by the Cut&Run assay. The remaining reads were mapped to the input viral genome using Bowtie2 (39), and the resulting SAM files were processed for identification and visualization of positional read counts using SAMtools (40).

### Sanger sequencing of KO cell lines.

We trypsinized 1 × 10^6^ HEK293 Scramble and KO cells, and they were then washed with PBS and spun down at 500 × *g* for 3mins to a pellet. The cell pellet was resuspended with fresh 200 μL PBS, and genomic DNA was isolated using the DNeasy blood and tissue kit (Qiagen) according to the manufacturer’s protocol. Primers binding 200 to 500 bp upstream and downstream of the sgRNA target sites were used to PCR amplify the indel region. PCR amplicons were gel purified and sequenced using the same set of primers.

### Statistical analysis.

Statistical analysis was performed with the GraphPad Prism software (v9.2) using two-tailed unpaired *t* test and ordinary one-way and two-way analyses of variance (ANOVAs). *P* values of <0.05 are considered significant and denoted in the figures as follows: *, *P < *0.05; **, *P < *0.01; ***, *P < *0.001; and ****, *P < *0.0001. Error bars represent standard deviation from the mean.
